# Estimation of Electrically-Evoked Knee Torque from Mechanomyography Using Support Vector Regression

**DOI:** 10.3390/s16071115

**Published:** 2016-07-19

**Authors:** Morufu Olusola Ibitoye, Nur Azah Hamzaid, Ahmad Khairi Abdul Wahab, Nazirah Hasnan, Sunday Olusanya Olatunji, Glen M. Davis

**Affiliations:** 1 Department of Biomedical Engineering, Faculty of Engineering, University of Malaya, Kuala Lumpur 50603, Malaysia; khairi@um.edu.my; 2 Department of Biomedical Engineering, Faculty of Engineering and Technology, University of Ilorin, P.M.B 1515, Ilorin 24003, Kwara State, Nigeria; 3 Department of Rehabilitation Medicine, Faculty of Medicine, University of Malaya, Kuala Lumpur 50603, Malaysia; nazirah@ummc.edu.my; 4 Computer Science Department, College of Computer Science & Information Technology, University of Dammam, Dammam 34212, Saudi Arabia; oluolatunji.aadam@gmail.com; 5 Clinical Exercise and Rehabilitation Unit, Discipline of Exercise and Sports Sciences, Faculty of Health Sciences, The University of Sydney, Sydney, 2006 NSW, Australia; glen.davis@sydney.edu.au

**Keywords:** support vector regression, gaussian kernel function, muscle force, mechanomyography, neuromuscular electrical stimulation, knee extension torque, regression model

## Abstract

The difficulty of real-time muscle force or joint torque estimation during neuromuscular electrical stimulation (NMES) in physical therapy and exercise science has motivated recent research interest in torque estimation from other muscle characteristics. This study investigated the accuracy of a computational intelligence technique for estimating NMES-evoked knee extension torque based on the Mechanomyographic signals (MMG) of contracting muscles that were recorded from eight healthy males. Simulation of the knee torque was modelled via Support Vector Regression (SVR) due to its good generalization ability in related fields. Inputs to the proposed model were MMG amplitude characteristics, the level of electrical stimulation or contraction intensity, and knee angle. Gaussian kernel function, as well as its optimal parameters were identified with the best performance measure and were applied as the SVR kernel function to build an effective knee torque estimation model. To train and test the model, the data were partitioned into training (70%) and testing (30%) subsets, respectively. The SVR estimation accuracy, based on the coefficient of determination (*R*^2^) between the actual and the estimated torque values was up to 94% and 89% during the training and testing cases, with root mean square errors (RMSE) of 9.48 and 12.95, respectively. The knee torque estimations obtained using SVR modelling agreed well with the experimental data from an isokinetic dynamometer. These findings support the realization of a closed-loop NMES system for functional tasks using MMG as the feedback signal source and an SVR algorithm for joint torque estimation.

## 1. Introduction

The magnitude of the muscle force or joint torque generated during neuromuscular electrical stimulation-evoked contractions has been used as a marker of physical performance in healthy individuals [1,2], as well as a benchmark of functional recovery in individuals with neurological conditions [3,4]. To optimize neuromuscular electrical stimulation (NMES) technology in therapeutic and functional applications, real-time information about the generated muscle force or joint torque, of the controlled limb, is vital [3,5]. Such information is required; (i) to automate the neuromuscular stimulation characteristics based on the muscle state during the onset of fatigue; and (ii) to modulate muscle forces based on the requirements of the task (therapeutic or functional) to be performed, for example during sit-to-stand and sustained standing perturbations. However, joint torque is often impractical or impossible to quantify directly during real-time application of NMES [5]. Estimation of joint torque from readily available muscle characteristics (e.g., biopotentials of nerve and/or muscle activation), particularly, from physical sensors has recently become both viable and attractive [5].

One such neuromuscular biopotential is the Mechanomyographic signal (MMG), which quantifies the mechanical equivalent of an electromyographic output generated during muscle contractions [6,7]. The signal originates from the skeletal muscle contractions due principally to the shortening of the muscle fiber length and increase in its diameter [7,8]. The activation of muscle fibres and their dimensional changes during muscle contraction creates pressure waves that can be detected on the skin surface and translated into an acceleration obtained by physical sensors, such as an accelerometer [9]. The signal can represent a proxy for neuromuscular contractions [10] and has gained recent popularity due to its close relationship with muscle force [11]. Specifically, the signal is directly related to the two main force-generating mechanisms of human skeletal muscle—magnitude and pattern of motor unit recruitment and their firing rates/frequency [12,13].

Moreover, due to the convenience of MMG signal collection, its insusceptibility to skin impedance [14], flexibility of its sensing technology [15,16], and immunity from electrical stimulation artifacts associated with NMES [17], the signal has been successfully used to classify muscle activity for specific application in controlling prostheses [18], and as a control signal for muscle machine interfaces [16,19]. In addition, during NMES-evoked muscle contractions, MMG has been used to track muscle fatigue in healthy volunteers [20]. Thus, the signal may be used to estimate joint torque during voluntary and/or NMES-evoked muscle contractions [7]. However, relating MMG signals as a direct proxy for NMES-evoked muscle effort/force can be practically challenging due to the complexity and diversity of the recruitment of muscle’s motor units (MUs) [6,18]. Accordingly, the application of computational intelligence techniques for quantification of joint torque from MMG signals has been proposed through statistical predictive modelling, and then validated during voluntary contractions [13,21].

The use of machine-learning techniques has recently shown promise, subverting the dual problems of non-linearity and non-stationarity in estimation, prediction and classification tasks. For example, Youn and Kim [13,22] used an artificial neural network model to estimate elbow flexion force from MMG during voluntary isometric contractions. The investigators obtained an estimation accuracy of up to 0.892 [13] and 0.883 [22] in terms of cross-correlation coefficient, and concluded that their model is subject dependent, while suggesting the future application of other machine learning techniques including Support Vector Regression (SVR) to improve the estimation accuracy of the model [22]. However, due to the advancement in the field of signal processing, several other computational intelligence statistical regression techniques have been proposed with SVR yielding a good predictive and estimation accuracy, with often low Root Mean Square Errors (RMSE) [23] and outstanding performance [21]. Being a category of support vector machine learning technique, SVR is based on the principles of computational intelligence that is built on the kernel method (that maps data into higher dimensional space where the training sample may be linearly separable to facilitate linear regression analysis) [24]. SVR algorithms take into account the error approximation to a dataset with the ability to adapt and improve the estimation capability of a model [23], particularly when the model is used to evaluate an additional dataset for the purpose of generalization [25,26]. Moreover, SVR is robust in handling multivariate processes and offsets the limitation of traditional regression methods [27]—which cannot solve problems with high dimensional input dataset [24]. Additionally, the SVR modelling only involves a solution to a “convex optimization problems”, and unlike ANN model, it is not influenced by the “local minimal problem” [28]. Thus, the SVR algorithms could be used to build a generalized model and well suited for regression tasks [24]. Based on this strength, the technique has been successfully deployed in several fields of applications including physical therapy and exercise science during voluntary muscle activation [21], medical diagnosis [29], and a host of other related fields. However, to our knowledge, SVR modelling has not been previously used to construct a joint torque estimation model, particularly, during electrically stimulated contraction. 

The purpose of this study was, therefore, to use SVR modelling to predict knee extensor joint torques from MMG signal characteristics during NMES-evoked incremental muscle contraction intensities. Since it has been suggested [13] that a combination of muscle contraction signals and related characteristics could compliment the estimation accuracy of joint torques, three input parameters (related to muscle contractions) to the SVR model were chosen (MMG signals, level of electrical stimulation or contraction intensity, and knee angle) to estimate knee torque accurately. This information is particularly applicable to research areas where a real-time proxy of muscle force is sought.

## 2. Materials and Methods

### 2.1. Experimental Protocol

**Figure 1:**
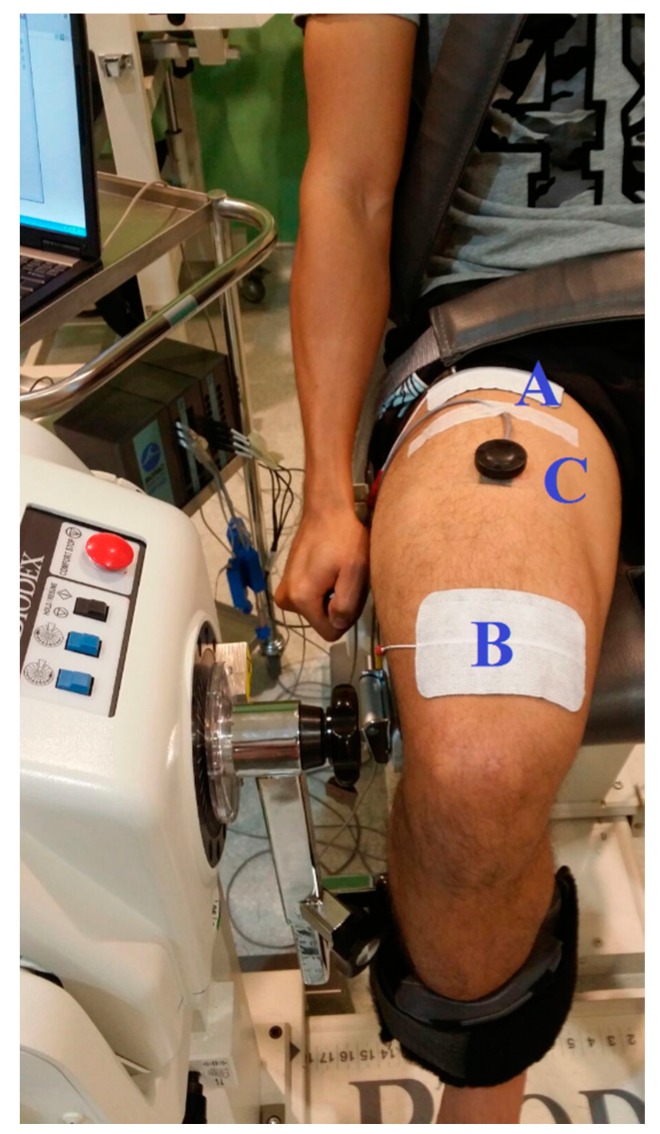
Experimental set-up at 90° knee angle showing the arrangement of stimulation electrodes (**A**) cathode, (**B**) anode Neuromuscular Electrical Stimulation (NMES) electrodes, and (**C**) Mechanomyographic signal (MMG) sensor in a representative participant.

To validate the performance of the proposed SVR model, a calibrated commercial dynamometer (System 4; Biodex Medical System, Shirley, NY, USA) was used to record isometric knee torques produced by NMES-evoked contractions of the knee extensors (Figure 1). Eight healthy male volunteers aged 23.4 (1.3) year (mean (SD)), body mass 70.4 (5.9) kg and height (1.72 (0.05)) m participated in this experiment. All were in good physical condition and were duly informed about the study protocol prior to giving their written informed consent. The study was approved by the University of Malaya Medical Ethics Committee (Approval No: 1003.14 (1)). As portrayed in Figure 1, the participants were set-up, as has been previously described by Brown and Weir [30] for voluntary isometric knee torque measurements. The dynamometer seat was adjusted so that each participant's lateral femoral condyle was aligned with the axle of the dynamometer [31]. To ensure consistency of body position and dynamometer lever arm, for subsequent trials, notes were taken of each participant’s relevant anatomical positions.

#### 2.1.1. NMES-Evoked Muscle Contractions and Knee Torque Measurements

A familiarization session (mimicking the actual test) preceded data collection to familiarize the participants to the study protocol and to habituate them to NMES-evoked knee extensors muscle contractions of maximally tolerable intensity. Thereafter, neuromuscular stimulation of square-wave pulses at 30 Hz frequency and 400 μs pulse duration, and incremental current amplitude from 20 mA to 80 mA (in 10 mA increments; i.e., seven different intensities of NMES or trial levels) was administered to elicit isometric torque of the knee extensors lasting 4 s [32]. Stimulation pulses were delivered through a commercially available computer-controlled neurostimulator (RehaStimTM, Hasomed GmbH, Magdeburg, Germany) using 9 × 15 cm^2^ self-adhesive electrodes (Hasomed GmbH, D 39114, Magdeburg, Germany) on the dominant leg [33]. To preclude voluntary effort, the participants were carefully instructed not to assist or resist NMES-evoked muscle contractions. A similar stimulation protocol has been used for strength training with tolerable discomfort [34] and without eliciting rapid muscle fatigue [35]. During each trial, the NMES-evoked torque at maximum stimulation intensity (80 mA) was taken as the NMES-evoked peak torque (PT). The PT value was used to normalize the submaximal contraction levels across participants’ data. The adopted stimulation electrode position has been recommended by Levin and colleagues [36]—the anode electrode placed at “~5 cm proximal position to the patella and the cathode electrode at ~8 cm distal to the inguinal area over the rectus femoris (RF) muscle belly near the expected location of the motor points” (Figure 1). In order to accommodate the effect of joint angle on joint torque [34,37], the experiment was conducted at three different randomized knee angles: 30°, 60°, and 90° (where 0° represented full knee extension). A duration of 48 h was allowed between each angle position, and there was a 10 min recovery between each trial to minimize potential muscle fatigue.

#### 2.1.2. MMG Acquisition and Processing

**Figure 2:**
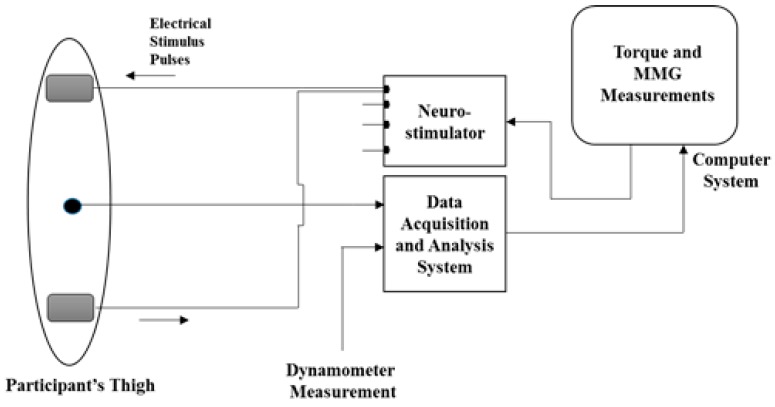
Schematic representation of the experimental setup. Stimulation electrodes (**A**) cathode, (**B**) anode NMES electrodes, and (**C**) MMG sensor.

Simultaneous with the NMES-evoked muscle contraction, MMG signals were collected using an accelerometer-based sensor (Sonostics BPS-II VMG transducer, sensitivity 30 V/g). The sensor was attached directly on the muscle belly (i.e., at the midpoint between the inguinal crease and the superior border of the patella [38] (Figure 1 and Figure 2) by means of double-sided adhesive tapes (3M Center St. Paul, MN, USA). The MMG signals were collected from the RF muscle as a simple representation of the knee extensors and a major contributor to the NMES-evoked knee torque production [39]. The signals were collected at 2 kHz sampling frequency and were digitally band-pass filtered at 20–200 Hz, amplified and stored by AcqKnowledge data acquisition and analysis software (MP150, BIOPAC Systems Inc., Santa Barbara, CA, USA) for offline analysis in the LabVIEW software environment (version 12.0, National Instruments, Austin, TX, USA) using custom written programs.

The peak torque values, MMG-root mean square (RMS) and peak to peak (PTP) amplitudes were obtained during NMES-evoked isometric contractions from 2 s epoch of the 4 s MMG and torque recordings [40] at each contraction level across the three joint angles. The selected 2 s epoch of the signals coincided with the middle position at which there was probable maximum muscle recruitment, without on-transients or off-transients of force rise at the beginning and the end of muscle contractions, respectively [40]. 

Thereafter, the MMG signals at each contraction level were normalized (by the equivalent value of the MMG signal at the highest stimulation intensity/contraction level (80 mA)) and fed into the proposed SVR model for training. Previous investigations [6,12] have validated the legitimacy of these MMG features for muscle force assessment, and, therefore, they were equally used as joint torque predictors in this study. The equations used to compute the MMG-RMS and MMG-PTP features [15] are as follows:

(1) $$\text{MMG}-\text{RMS}=\sqrt{\frac{1}{N}\sum_{i=1}^{N}x_{i}^{2}}\;\mathrm{where}\;\sum_{i=1}^{N}x_{i}^{2}\;=x_{1}^{2}+x_{2}^{2}+x_{3}^{2}+\;\ldots +x_{N}^{2})$$

(2) $$\text{MMG}-\text{PTP}=\;2\sqrt{2}\;\text{RMS}$$

here, $x_{i}$ is the ith sample of the MMG signals and N represents the number of samples in the epoch considered.

### 2.2. Support Vector Regression Modelling Approach

SVR algorithm was proposed in this study because of its optimal predictive performance even with small dataset [41] and the ability to learn both linear and non-linear relationships between predictors and outcome (actual) variables [42]. Such relationships have been used in establishing a pattern whereby unknown outcomes could be predicted accurately [21,24]. Theoretically, SVR is derived from the statistical learning theory [43,44] and employs ε-insensitive loss function [24] which measures the flatness of the generated pattern as well as maximum allowable deviations of the targets from the predicted values for all given training datasets $\left(x_{1},y_{1}),..........,(x_{k},y_{k}\right)$ with k number of samples [45]. However, a function used for the SVR analysis should not only approximate the training data adequately but also predicts accurately the value of y for the future data x [25]. Such a function, with 〈w,x〉 dot product in the space of $R^{\prime }$, is represented in linear form by Equation (3) for a set of training samples.

(3) f(x,α)=〈w,x〉+b

where $w\in R^{\prime }$ and b∈R

For the purpose of establishing the goal of SVR in ensuring the flatness of the Equation (3), small value of w is desired through minimization of the Euclidean norm ${\left\|w\right\|}^{2}$ [42] which makes the optimization problem of the regression to be represented by Equation (4):

(4) $$\begin{array}{l} \text{minimize}\;\frac{1}{2}{\left\|w\right\|}^{2}\\ subject\;to\left\{\begin{array}{l} y_{i}-\langle w,x_{i}\rangle -b\leq \varepsilon \\ \langle w,x_{i}\rangle +b-y_{i}\leq \varepsilon \end{array}\right\} \end{array}$$

Equation (4) holds on the assumption [46] that there exists a function that is capable of providing error which is less than ε for all training pairs of the dataset. The slack variables $\left(\xi_{i}\;\text{and}\;{\xi^{*}}_{i}\right)$, which represent the upper and lower constraints on the system output, are often introduced in order to permit some errors that are associated with real life problems [44,46]. Therefore, Equation (4) is modified and presented as Equation (5).

(5) $$\begin{array}{l} \text{minimize}\;\frac{1}{2}{\left\|w\right\|}^{2}+C\sum_{i=1}^{k}\left(\xi_{i}+{\xi^{*}}_{i}\right)\\ subject\;to\left\{\begin{array}{l} y_{i}-\langle w,x_{i}\rangle -b\leq \varepsilon +\xi_{i}\\ \langle w,x_{i}\rangle +b-y_{i}\leq \varepsilon +{\xi^{*}}_{i}\\ \xi_{i},{\xi^{*}}_{i}\geq 0\;\;\text{for}\;\text{all}\;i=1,2,......,k \end{array}\right\} \end{array}$$

The optimization problem in Equation (5) is better solved, through the ε-insensitive loss function, by using Lagrangian multipliers ($\eta_{i},\eta_{i}^{*},\lambda_{i}\;\text{and}\;\lambda_{i}^{*}$) to transform the problem into dual space representation. Therefore, the Lagrangian for the Equation (5) is presented in Equation (6).

(6) $$\begin{array}{l} L=\frac{1}{2}{\left\|w\right\|}^{2}+C\sum_{i=1}^{k}\left(\xi_{i}+\xi_{i}^{*}\right)-\sum_{i=1}^{k}\lambda_{i}\left(\varepsilon +\xi_{i}-y_{i}+\langle w,x_{i}\rangle +b\right)\\ -\sum_{i=1}^{k}\lambda_{i}^{*}\left(\varepsilon +\xi_{i}^{*}+y_{i}-\langle w,x_{i}\rangle -b\right)-\sum_{i=1}^{k}\left(\eta_{i}\xi_{i}+\eta_{i}^{*}\xi_{i}^{*}\right) \end{array}$$

It is easier to locate the saddle point of the Lagrangian function defined in Equation (6) by equating the partial derivatives of the Lagrangian $\left(\text{with}\;\text{respect}\;\text{to}\;w,b,\xi_{i}\;\text{and}\;\xi_{i}^{*}\right)$ to zero in order to obtain the expressions presented in Equations (7)–(9):

(7) $$w=\sum_{i=1}^{k}\left(\lambda_{i}^{*}-\lambda_{i}\right).x_{i}$$

(8) $$\eta_{i}=C-\lambda_{i}$$

(9) $${\eta^{*}}_{i}=C-\lambda_{i}^{*}$$

The optimization equation is maximized by substituting Equations (7)–(9) in Equation (6) to arrive at Equation (10):

(10) $$\begin{array}{l} \text{maximize}\;\frac{1}{2}\sum_{i=1}^{k}\sum_{j=1}^{k}\left(\lambda_{i}^{*}-\lambda_{i}\right)\left(\lambda_{j}^{*}-\lambda_{j}\right)\left(x_{j}.x_{i}\right)-\varepsilon \sum_{i=1}^{k}\left(\lambda_{i}^{*}+\lambda_{i}\right)+\sum_{i=1}^{k}y_{i}\left(\lambda_{i}^{*}-\lambda_{i}\right)\\ subject\;\text{to}\;\sum_{i=1}^{k}\left(\lambda_{i}^{*}-\lambda_{i}\right)=0,0\leq {\lambda^{*}}_{i}\;\text{and}\;\lambda_{i}\leq C \end{array}$$

The solutions ($\lambda_{i}^{*}\;\text{and}\;\lambda_{i}$) obtained from Equation (10) are substituted in Equation (3) and presented in Equation (11):

(11) $$f(x,\alpha )=\sum_{i=1}^{k}\left(\lambda_{i}^{*}-\lambda_{i}\right)\langle x_{i},x\rangle +b$$

However, since the concept of kernel function through ‘’kernel tricks’’ allows SVR to solve non-linear problems by mapping the original non-linear data into higher dimensional feature space where a linear model could be constructed [47], a proper selection of kernel function allows optimization of SVR performance [47]. The regression function in feature space, after inserting the kernel function $K\langle x_{i},x\rangle$, could be written as presented in Equation (12).

(12) $$f(x,\alpha )=\sum_{i=1}^{k}\left(\lambda_{i}^{*}-\lambda_{i}\right)K\langle x_{i},x\rangle +b$$

Kernel functions help in transforming datasets into hyperplane [47]. The variables of the kernel function determine the structure of high dimensional feature space which controls the complexity of the final solution. As applied in this study, Equations (13)–(16) describe several kernel functions that are obtainable in the literature [48] which include Polynomial, Linear, Gaussian (radial basis function (RBF)) and Sigmoid.

(13) $$K(\vec{x_{i}},\vec{x_{j}})={\left(\vec{x_{i}}\cdot \vec{x_{j}}+1\right)}^{d}$$

(14) $$K(x_{i},x_{j})={x_{i}}^{T}.x_{j}$$

(15) $$K(\vec{x_{i}},\vec{x_{j}})=\exp\left(-\gamma {\left\|\vec{x_{i}}-\vec{x_{j}}\right\|}^{d}\right)$$

(16) $$K(x_{i},x_{j})=\tanh(\gamma {x_{i}}^{T}x_{j}+r)$$

where γ,r,and d are kernel parameters and, $\vec{x_{i}}$ and $\vec{x_{j}}$ represent vectors in the input space—vectors of features computed from training or test subset [23].

#### 2.2.1. Model Development

MATLAB software environment (Version 12, The MathWorks, Inc., Natick, MA, USA) using SVR coding was used for the computational aspect of this research work. Prior to the use of the dataset, the dataset was partitioned into two components to adhere to the SVR modelling approach [23,49]—a machine-learning “training” subset and a “testing” subset in a ratio of 7:3, via stratified sampling to ensure effective random partitioning [50]. Specifically, 70% of the dataset was used for training and the remaining 30% was used for testing the SVR model via test-set cross-validation method. This allowed a regression analysis to be performed on the training dataset while estimating the future generalization accuracy, of the model, on the remaining testing subset. For further detail on the working principle of the proposed SVR model, readers are referred to Vapnik et al. (1997) [24], Lin S-W et al. (2008) [47], Shamshirband et al. (2014) [23], and Akande et al. (2015) [49].

#### 2.2.2. Optimal Parameters Search Approach

**Figure 3:**
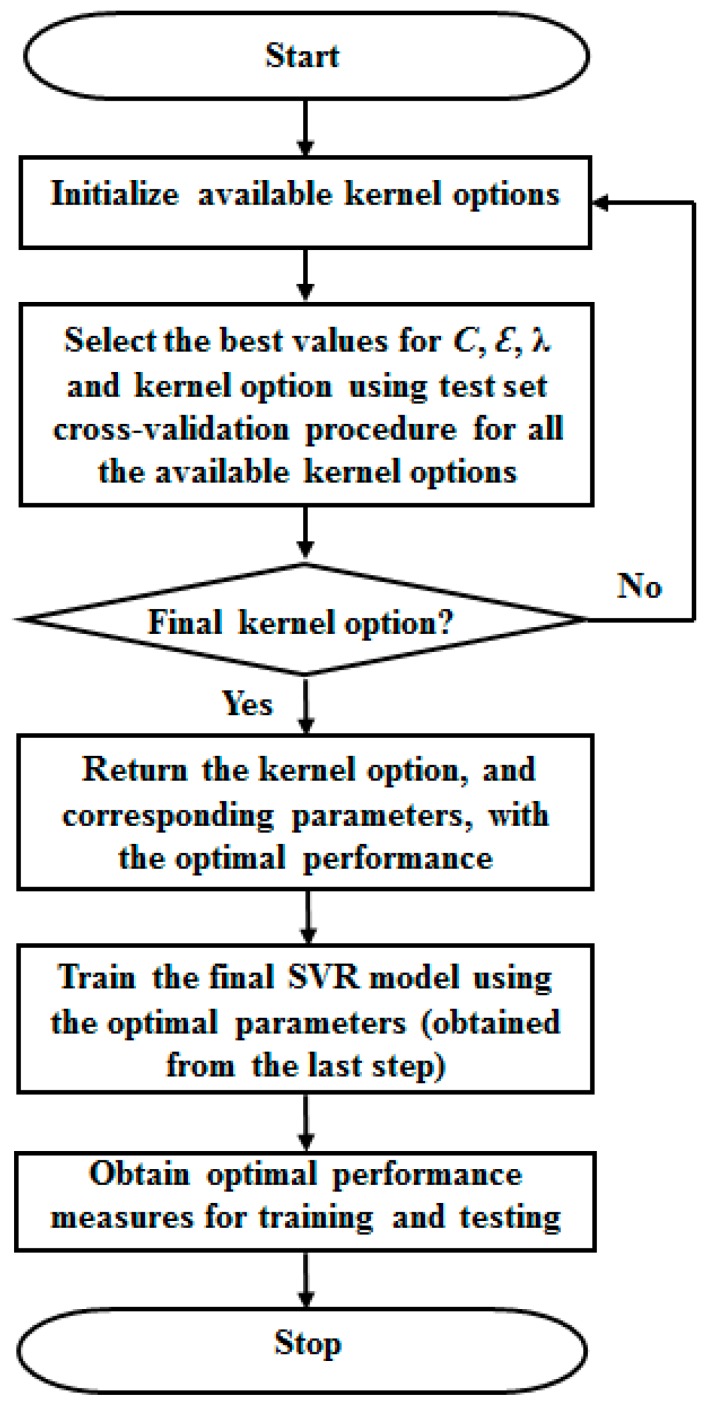
Flow chart of the procedure for obtaining optimal parameters (Table 1) for the proposed SVR model.

The accuracy of a SVR model is dependent on the model parameters’ selection [23]. However, due to the possibility of many different combinations of SVR parameters, it is often difficult to obtain optimal SVR parameters [51]. To solve this problem systematically, and in order to obtain possible optimized parameters of SVR for an accurate estimation, a hybrid optimization search technique, which has been recommended [52], was adopted and a test-set cross-validation technique was deployed [53]. The approach is as follows: for every partitioned training and testing subsets, the performance measures were noted for the SVR parameters values including the regularization factor *C* (bound on the Lagrangian multiplier), λ (conditioning parameter for quadratic programming (QP) methods), ε (epsilon) and η (kernel option) as well as the related kernel functions [49]. Thereafter, this computational step was repeated for every available SVR kernel function with an incremental step of the parameters’ values. The parameters’ optimal values and the kernel function associated with the best performance measure were identified. The search procedures are presented summarily in Figure 3.

A mathematical implementation [49] of the test-set cross-validation technique is as described in Algorithm 1 as follows: $K_{i}\left(j\right)$ was defined where K contains all the available kernel functions (and i, j and k are the indexes for the kernel functions) while iy, jy and ky represent the indexes for optimal kernel function. The total number of the available kernel function is represented by ni. The maximum values of C and η were assumed to be nj and nk, respectively. The recorded performance measures were stored in pf.

**Algorithm 1.** Optimal parameter search algorithmInitialization;
iy=0, jy=0, ky=0, qx=0 for i=1:ni for j=1:nj
$pf=f\left(K_{i}\left(j\right)\right)$ for k=1:nk $pf=f\left(K_{i}\left(j\right)\right)$ {Performance measure for the present parameters combination} if pf is better than qx then qx=pf iy=i, jy=j, ky=k {storing the index of the best parameter} *end* *end* *end*

#### 2.2.3. Model Statistical Performance Criteria

To evaluate the performance of the proposed model, common measures of association, between the actual and the estimated values, were employed, including correlation coefficient (*r*) and coefficient of determination (*R*^2^) to quantify the “goodness of fit’’, and Root Mean Square Error (RMSE) to quantify the error of estimate. For further details on their mathematical formulae, readers are referred to Youn and Kim (2011) [22] and Olatunji et al. (2014) [54].

## 3. Results and Discussion

Table 2 describes the actual experimental dataset used in this study. The results of the statistical analysis of the dataset are presented in Table 3. The suitability and applicability of the chosen dataset are revealed from the mean, maximum value, median, standard deviation, and minimum value. The MMG-RMS, MMG-PTP, level of electrical stimulation or contraction intensity, and knee angle obtained experimentally were the input to the SVR model to estimate the knee torque. Results of performance measures obtained from the training subset and testing subset are as shown in Table 4. 

To our knowledge, this is the first attempt to use a SVR modelling technique for NMES-evoked knee torque estimation from MMG signal. The outcomes of the developed SVR model (Table 4) indicated high correlation as well as low RMSE, and the model could, therefore, be adjudged as accurate. Moreover, high accuracy of the trained system as evident by the coefficient of determination (*R*^2^ = 94%), in predicting knee torque confirmed a reliable pattern between the predictors and the outcome which might be otherwise difficult to learn using linear regression.

**Figure 4:**
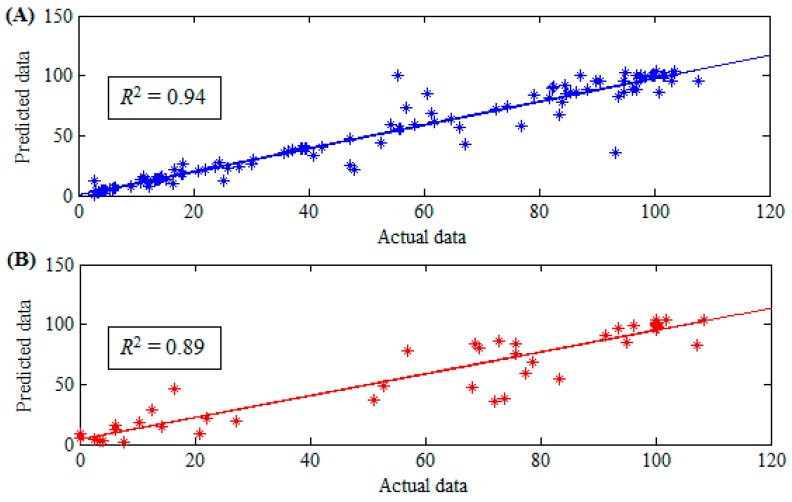
Plots of the correlation coefficients for the training (**A**) and testing; (**B**) subsets.

During the training period of the model, the estimated torques were positively correlated with the actual values drawn from the experimental data (actual vs. predicted values) for both the training (Figure 4A) and testing (Figure 4B) subsets.

**Figure 5:**
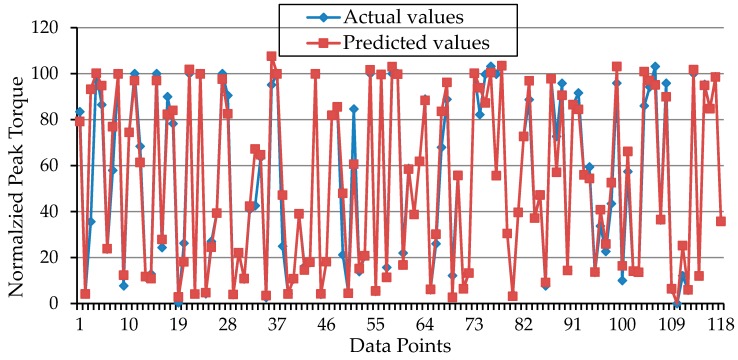
Cross plots of training sets—actual vs. predicted values: The plots show the performance of SVR with Gaussian kernel for torque prediction on the training set.

In addition, the cross-plots of the “training’’ subsets (actual vs. predicted values) as shown in Figure 5 also confirmed the high accuracy of the “training” subsets. However, since the actual performance of any model is better accessed by the testing outcome [55], the accuracy of the developed SVR model was tested using 30% of the available data samples (i.e., the reserved 30% that was not used in model development). It was interesting to note that, the model also performed satisfactorily during testing phase (*R*^2^ = 89%).

**Figure 6:**
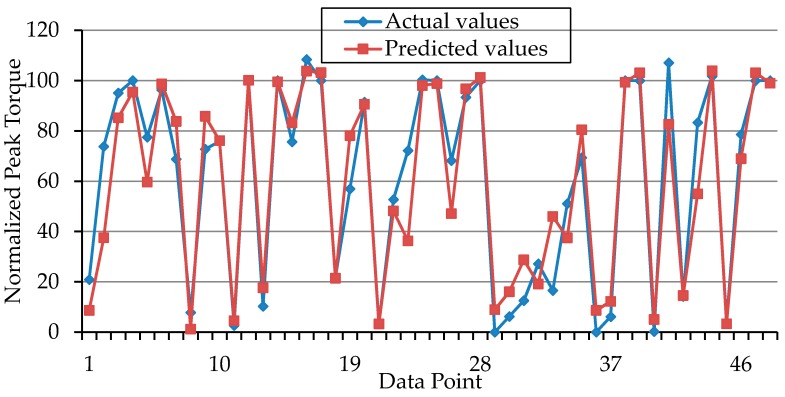
Cross plots of testing set sets—actual vs. predicted values: The plots show the performance of SVR with Gaussian kernel for torque prediction on the testing set.

This high correlation indicated that the estimated knee torque by the SVR model was very close to the actual experimentally recorded joint torque (from an isokinetic dynamometer) for each data sample. For better visualization and understanding of the outcome of this study, the cross-plot of testing sets (actual vs. predicted values) has been portrayed in Figure 6. The level of accuracy in the testing phase (*R*^2^ = 94%) of the model development indicates that the model is stable, efficient and not over-fitted. This was based on the suggestion of Tay and Cao [56] that an overfitted model could perform excellently on the training set (*r* > 0.90) but will perform poorly on testing set [56]. Therefore, the developed SVR model in this study achieved a good performance for both training and testing sets. These results are comparable to that of Youn and Kim [22], where an artificial neural network model has been successfully used to estimate elbow force during voluntary contractions. Meanwhile, the potential of the SVR model for NMES-evoked joint torque estimation which has not been previously documented has also been demonstrated in our study.

Moreover, Figure 5 and Figure 6 portrayed the closeness of the predicted torque by the proposed SVR model to the actual experimental values. It could be noted that almost all the predicted points fit exactly on the experimental point or at least fits very closely to the target experimental point. Taken together, it could be inferred that the real-time knee torque information which is vital for the closed-loop implementation of NMES [3,5] in physical therapy and exercise science might be reliably estimated by our proposed method. Nevertheless, we acknowledge the following limitation in our study design: The performance of the developed model is limited to torque estimation during NMES-evoked isometric knee extension in healthy volunteers. In the future studies, we will verify the performance of the model using MMG signal and torque data from participants with neurological conditions. This will allow us to examine and improve the performance of the model, and to derive clinically relevant characteristics about the muscle force recruitment in clinical populations.

## 4. Conclusions

Based on its previous estimation accuracy in relevant fields, SVR modelling was used in this study through the integration of relevant variables to predict NMES-evoked knee torque. The model was developed through training and testing via test-set cross-validation technique with available dataset partitioned into training and testing subsets. Using the SVR methodology, the predicted knee torque was positively correlated with the actual values drawn from the experimental data for the training subset. Thereafter, to check the predictive ability of the model, the trained model was tested using the reserved testing subset that was not used in model development. The model performance was measured based on the correlation coefficient and RMSE. The outcomes from the developed SVR model showed an accurate prediction of the knee torque, characterized by high correlation coefficient—up to 0.97 and 0.94; and coefficient of determination—up to 94% and 89%, and low RMSE of 9.48 and 12.95, for the training and testing cases, respectively. These results, which have not been previously reported, indicated a close similarity between the estimated joint torque by the SVR model and the actual experimental data obtained from the laboratory experiment. Additionally, the present study has uniquely shown that a SVR model could estimate NMES-evoked knee torque, generated by a synchronous modulation of muscle fibres’ motor units [57], from MMG signal. Therefore, the good performance achieved in this study will motivate further studies in a similar direction to facilitate accurate estimations of joint torque using datasets from clinical populations—in which the NMES technology is more relevant, particularly among those with spinal cord injury. Moreover, since SVR models can be adapted for classification tasks [43], in the future, the developed model will be used to classify fresh and fatiguing muscle contractions of knee extensors, from MMG signals, during standing and ambulation tasks. Such models might offset the need to contend with the stimulation artifact [3,5] often encountered with the application of surface electromyographic signal as NMES feedback source.

## Figures and Tables

**Table 1:** Optimal parameters for the proposed Support Vector Regression model.

*C*	879
Hyper-parameter (Lambda)	2^−15^
Epsilon (ε)	0.1205
Kernel option	54
Kernel	Gaussian (RBF)

**Table 2:** Summary of the datasets: Mechanomyographic signal (MMG) characteristics at seven Neuromuscular Electrical Stimulation (NMES) intensities, at three knee angles and their respective peak torque values.

Stimulation Intensity (mA)	Knee Angle								
	30°			60°			90°		
	PT	RMS	PTP	PT	RMS	PTP	PT	RMS	PTP
20	13.9 (3.7)	14.7 (9.9)	23.6 (16.4)	4.1 (0.7)	17.4 (18.4)	21.7 (23.0)	4.3 (5.7)	20.4 (20.0)	22.0 (26.9)
30	23.3 (19.7)	51.9 (22.4)	55.8 (24.7)	9.7 (8.5)	37.4 (21.2)	38.7 (19.5)	11.0 (10.2)	51.3 (30.0)	50.8 (30.6)
40	58.2 (23.6)	75.3 (29.5)	73.54 (19.1)	27.6 (24.2)	77.7 (36.6)	65.88 (19.2)	21.4 (15.0)	93.4 (45.4)	84.0 (34.1)
50	76.6 (19.3)	84.2 (15.2)	85.04 (14.9)	51.5 (26.2)	82.6 (27.3)	72.7 (14.9)	40.7 (18.5)	115.7 (39.6)	101.0 (33.8)
60	86.1 (20.2)	104.9 (22.5)	94.86 (18.2)	74.7 (19.2)	94.9 (30.4)	85.27 (14.7)	62.1 (12.3)	104.3 (29.0)	101.1 (28.5)
70	91.1 (21.5)	100.2 (6.2)	98.34 (5.7)	91.0 (8.2)	88.1 (9.7)	90.57 (14.4)	84.2 (12.3)	118.5 (22.0)	113.2 (10.7)
80	100.0 (0)	100.0 (0)	100.0 (0)	100.0 (0)	100.0 (0)	100.0 (0)	100.0 (0)	100.0 (0)	100.0 (0)

Abbreviations: Stimulation Intensity—level of electrical stimulation or contraction intensity, PT—Peak torque, RMS—Normalized MMG-RMS%, PTP—Normalized MMG-PTP%. Values are reported in mean (standard deviation) for N = 8.

**Table 3:** Statistical parameters of the datasets.

Input Parameters	Mean	Max	Median	Stdev	Min
Participants					
Weight (kg)	70.1	80	69	5.9	63
Age (years)	23.4	25	23.5	1.3	21
Stimulation intensity (mA)	50	80	50	20	20
Knee angle (°)	60	90	60	24.5	30
Normalized MMG-RMS%	77.8	188.1	86.9	40.0	4
Normalized MMG-PTP%	75.2	163.5	81.6	34.8	4.6
Peak torque	53.9	108.4	57.2	38	0

**Table 4:** Performance measures that determined the accuracy of the developed model.

Performance Measures	Training	Testing
*r*	0.97	0.94
*R*^2^	94%	89%
RMSE	9.48	12.95

## Abbreviations

The following abbreviations are used in this manuscript:

NMES	Neuromuscular Electrical Stimulation
MMG	Mechanomyography
SVR	Support Vector Regression
SVM	Support Vector Machine
RMSE	Root Mean Square Error
PT	Peak Torque
RF	Rectus Femoris
RMS	Root Mean Square
PTP	Peak to Peak
